# T69. PERFORMANCE OF PATIENTS WITH NMDAR-AB ENCEPHALITIS COMPARED TO PATIENTS WITH SCHIZOPHRENIA AND HEALTHY CONTROLS USING FUNCTIONAL MAGNETIC RESONANCE IMAGING (F-MRI) AND A WORKING MEMORY PARADIGM

**DOI:** 10.1093/schbul/sby016.345

**Published:** 2018-04-01

**Authors:** Eric Kelleher, David Mothersill, April Hargreaves, Angela Vincent, Gary Donohoe, Aiden Corvin

**Affiliations:** 1 Trinity Centre for Health Sciences; 2 National University of Ireland; 3 Trinity College Dublin; 4 University of Oxford

## Abstract

**Background:**

N-methyl-D-aspartate receptor (NMDAR) encephalitis has prominent early psychotic features. It has been rarely misdiagnosed as schizophrenia. NMDAR encephalitis causes deficiencies in working memory in the short & long term. Early immunotherapy/tumour removal improves outcomes. Schizophrenia can cause significant cognitive performance deficits with working memory often severely affected.

Clinical MRI in NMDAR encephalitis is usually normal, despite severe clinical course. Resting state fMRI studies have shown reduced functional connectivity of the left & right hippocampus. To date, to the best of our knowledge, functional neuroimaging utilising a working memory task such as the 1-back task (participant has to identify the number onscreen immediately before the number currently on screen) has not been utilised in NMDAR encephalitis. Potentially this may highlight alterations in brain regions through altered neuronal activity underlying working memory.

We hypothesised that patients with NMDAR encephalitis would demonstrate a difference in BOLD response (a proxy for neuronal activity) compared to healthy controls during fMRI while performing the 1-back task. Additionally, we wished to compare task evoked BOLD response in patients with NMDAR-Ab encephalitis compared to patients with schizophrenia.

**Methods:**

Using G*Power 3.1 software with effect size f (0.8), total sample size was calculated from data obtained from a previous study on working memory performance in NMDAR encephalitis for the 3 groups (NMDAR encephalitis, healthy controls & schizophrenia) (N=30). Following ethical approval, 12 individuals diagnosed with NMDAR encephalitis were recruited from neurological centres. Participants were trained in the 1-back task prior to scanning. Results were compared to previously collected data from cohorts of healthy controls (N=14) and schizophrenia (N=14). Cohorts were matched insofar as possible. Data was analysed using SPSS and ART toolbox.

**Results:**

In the NMDAR encephalitis (N=12) cohort, gender ratio F:M was (11:1), mean(s.d.) age 37.5(12.2) years. 50% identified ongoing memory problems. Schizophrenia cohort (N=14) gender ratio (7:7), mean (s.d.) age 39 (12.3) years. Healthy controls gender ratio (10.4) mean (s.d.) age 29.8 (6.5) years. There was no difference for gender, age or educational level between cohorts. FSIQ was higher (p<0.01) in controls & NMDAR encephalitis compared to schizophrenia cohorts. NMDAR encephalitis cohort had acute symptoms [mean (s.d.)] for 7.09 (2.43) weeks until treatment. 9 individuals were still on immunotherapy at time of entry.Duration since diagnosis to entry into this study was 55.18(23.13) months for NMDAR encephalitis and 12.6 (10.91) years for schizophrenia. There were no significant differences in number of ART motion outliers (p >0.05) between cohorts. Age & gender were added to second level analyses as covariates.

One-way ANOVA was used to compare N-back accuracy & reaction time. There was a significant difference (p<0.01) between groups on accuracy but not reaction time, with only the schizophrenia group showing lower accuracy.1-back working memory condition was associated with significantly increased BOLD response across several brain regions that play a role in working memory, including the dorsolateral prefrontal cortex. However, there were no significant main effects on BOLD response for the 1-back task for any cohort.

**Discussion:**

Patients with NMDAR encephalitis report ongoing memory difficulties. However, in this study, there were no identifiable deficits compared to healthy controls using fMRI. This may relate to regeneration of NMDA receptors, the relatively long time until from diagnosis to follow up and early treatment with immunotherapy.

